# Genes and Pathways Associated with Skeletal Sagittal Malocclusions: A Systematic Review

**DOI:** 10.3390/ijms222313037

**Published:** 2021-12-02

**Authors:** Elizabeth Gershater, Chenshuang Li, Pin Ha, Chun-Hsi Chung, Nipul Tanna, Min Zou, Zhong Zheng

**Affiliations:** 1 Department of Biology, Muhlenberg College, Allentown, PA 18104, USA; elizabethgershater@gmail.com; 2 School of Dental Medicine, Department of Orthodontics, University of Pennsylvania, Philadelphia, PA 19104, USA; chunc@upenn.edu (C.-H.C.); nipul77@upenn.edu (N.T.); 3 David Geffen School of Medicine, University of California, Los Angeles, CA 90095, USA; hapin1987@ucla.edu; 4 Key Laboratory of Shannxi Province for Craniofacial Precision Medicine Research, College of Stomatology, Xi’an Jiaotong University, 98 XiWu Road, Xi’an 710004, China; zoumin@mail.xjtu.edu.cn; 5 Clinical Research Center of Shannxi Province for Dental and Maxillofacial Diseases, College of Stomatology, Xi’an Jiaotong University, 98 XiWu Road, Xi’an 710004, China; 6 Department of Orthodontics, College of Stomatology, Xi’an Jiaotong University, 98 XiWu Road, Xi’an 710004, China; 7 School of Dentistry, University of California, Los Angeles, CA 90095, USA

**Keywords:** skeletal class II, skeletal class III, mandibular prognathism, mandibular retrognathism, malocclusion, bone, GWAS, SNP, functional matrix theory

## Abstract

Skeletal class II and III malocclusions are craniofacial disorders that negatively impact people’s quality of life worldwide. Unfortunately, the growth patterns of skeletal malocclusions and their clinical correction prognoses are difficult to predict largely due to lack of knowledge of their precise etiology. Inspired by the strong inheritance pattern of a specific type of skeletal malocclusion, previous genome-wide association studies (GWAS) were reanalyzed, resulting in the identification of 19 skeletal class II malocclusion-associated and 53 skeletal class III malocclusion-associated genes. Functional enrichment of these genes created a signal pathway atlas in which most of the genes were associated with bone and cartilage growth and development, as expected, while some were characterized by functions related to skeletal muscle maturation and construction. Interestingly, several genes and enriched pathways are involved in both skeletal class II and III malocclusions, indicating the key regulatory effects of these genes and pathways in craniofacial development. There is no doubt that further investigation is necessary to validate these recognized genes’ and pathways’ specific function(s) related to maxillary and mandibular development. In summary, this systematic review provides initial insight on developing novel gene-based treatment strategies for skeletal malocclusions and paves the path for precision medicine where dental care providers can make an accurate prediction of the craniofacial growth of an individual patient based on his/her genetic profile.

## 1. Introduction

Among the three dimensions of craniofacial structure, the sagittal dimension is the primary focus and most crucial plane in orthodontic diagnosis and treatment planning [1]. Skeletal class II and III malocclusions are disorders with a sagittal discrepancy between the maxilla and the mandible [2,3]: patients diagnosed with skeletal class II malocclusion exhibit an anterior position of the maxilla in comparison with the mandible, which could result from mandibular retrognathism (reduced lower jaw growth and size) and/or maxillary prognathism (excessive upper jaw growth and size) [2,4]. In contrast, skeletal class III malocclusion, characterized by a posterior maxilla position related to the mandible, could be attributed to mandibular prognathism, maxillary retrognathism, or a combination of the two [3,5]. Both skeletal class II and III malocclusions significantly diminish patients’ ability to chew food effectively [6]. In addition, patients with skeletal class III malocclusion have a relatively high level of abrupted, discontinuous masticatory motions and an impaired ability to swallow a bolus [7]. Skeletal class II and III malocclusions may also deleteriously affect the functions of other components of the digestive system [8]. Moreover, skeletal class II and III malocclusions have been linked to a diversity of maladies that concern oral health, such as myogenic temporomandibular disorders [9], and even extend to other parts of the body, such as myofascial pain [10] and gastroesophageal reflux disease [8]. Furthermore, from a cosmetic standpoint, skeletal class II and III malocclusions are known to be visually unappealing traits [11,12], which may also harm patients’ psychological well-being and decrease their perception of their own worth. In contrast, surgical correction could markedly increase their self-esteem and decrease anxieties about their visual appearance [11]. Together, skeletal class II and III malocclusions significantly negatively impact people’s quality of life [13]. Strikingly, approximately a quarter of the general population suffers from one or the other of these types of malocclusions [14]. Indeed, the global incidence of skeletal class II malocclusion is estimated at nearly 20% and that of skeletal class III malocclusion at 6% [14]. Therefore, treating these types of malocclusions and thereby rectifying patients’ dentition would tremendously benefit individuals and society.

To date, orthognathic surgical correction is still the last-resort treatment of severe skeletal class II and III malocclusions to achieve balanced anatomic and functional jaw relationships and improve facial appearance [15]. However, as an invasive procedure, orthognathic surgery is associated with various, sometimes severe, risks, including impaired sensation, infection, bleeding, tissue injury, and alterations in the osteosynthesis systems [16]. More importantly, to achieve proper diagnosis and avoid additional surgeries to correct malocclusion relapse due to post-surgery growth, most orthognathic surgeries can only be achieved when patients are skeletally matured [17]. Furthermore, the long-standing expectation of surgery during childhood and adolescence will worsen patients’ physical, social, and psychological health. In order to avoid surgical operations and the consequences associated with such treatments, a large amount of effort has been dedicated to developing growth modification appliances for skeletal class II and III malocclusion correction, especially those that can be delivered around the puberty stage to achieve the normal dentition without surgery [18,19,20,21]. Unfortunately, despite the tremendous efforts that have been devoted to developing a diversity of treatment technologies [11,18,19,20,21,22,23,24,25], dental care providers are not yet able to predict whether an orthodontic functional appliance will successfully correct a patient’s skeletal discrepancy or if that patient will not respond to early orthopedic correction and require surgery to properly rectify their malocclusion later. In other words, people’s expected development of skeletal malocclusion and their clinical correction prognoses are currently difficult to predict, which can be largely attributed to the present lack of knowledge of the precise etiology of skeletal class II and III malocclusions.

Since a strong inheritance pattern of a specific type of skeletal malocclusion running in families has been observed in various races [14,26], there is no doubt that genomic alteration(s) play essential roles in skeletal malocclusion development. Here, previous genome-wide association studies (GWAS) are summarized and reanalyzed to identify skeletal class II and III malocclusion-associated genes and signal pathways. Therefore, by discussing the genetic factors that may regulate the abnormal maxillary and mandibular growth that cause skeletal class II and III malocclusions, this systematic review aims to provide initial insight into developing novel gene-based treatment strategies for skeletal malocclusions and paves the path for precision medicine where dental care providers can make an accurate prediction of the craniofacial growth of an individual patient based on his/her genetic profile.

## 2. Methods

**Figure 1:**
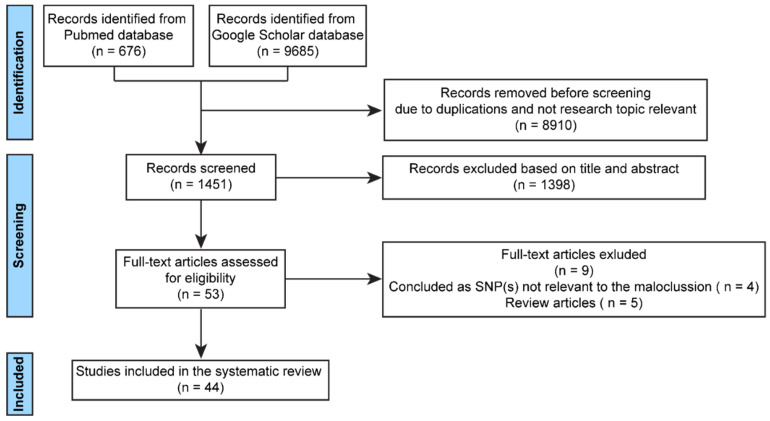
Flow diagram of the selection of articles referring to genes related to skeletal class II or skeletal class III malocclusions.

This systematic review followed the 2020 Preferred Reporting Items for Systemic Reviews and Meta-Analyses (PRISMA) guidelines [27], and the article search was conducted in June 2021. The PubMed and Google Scholar databases were searched using the following keywords: “gene”, “genetic”, “GWAS”, “mandible”, “mandibular”, “prognathism”, “retrognathism”, “skeletal class II”, and “skeletal class III”. No publication date limits were set. Reviews were excluded from consideration to avoid double-counting of genes, and only studies conducted on humans and written in English were included. Studies were evaluated independently by two authors (E. G. and C. L.) and were selected for use in the current systematic review if both authors determined that the studies were eligible (Figure 1). It is important to note that the genes found in the databases pertained to skeletal class II and III malocclusions, with some research specifically focused on a given gene’s association with the maxilla or mandible only. Because gene expression in the mandible and the maxilla can contribute to skeletal class II and III malocclusions, both potential sources of malocclusion were considered in this review.

Skeletal classes II or III malocclusion-associated genes were then separately entered into the Reactome database for pathway enrichment [28]. The pathways associated with each skeletal malocclusion with a *p*-value and a false discovery rate (FDR) less than 0.05 were recorded, and the top 20 pathways with the lowest FDR values were further discussed. Pathway functional annotations were based on PubMed focusing on bone, cartilage, osteoblasts, osteoclasts, and osteocytes. Functions of genes not recognized in the Reactome database were annotated by the Uniprot database [29].

## 3. Results

### 3.1. Skeletal Class II Malocclusion

#### 3.1.1. Gene Identification

Using the PubMed and Google Scholar databases, 15 human studies targeting skeletal class II malocclusion were collected. Nineteen skeletal class II malocclusion-associated genes were identified, among which five genes (*actinin alpha 3* (*ACTN3*), *ADAM metallopeptidase with thrombospondin type 1 motif 9* (*ADAMTS9*), *fibroblast growth factor receptor 2* (*FGFR2*), *msh homeobox 1* (*MSX1*), and *myosin IH* (*MYO1H*)) were investigated twice by previously conducted GWAS studies, and the other 14 genes once (Table 1).

#### 3.1.2. Pathway Enrichment

A total of 17 of these 19 skeletal class II malocclusion-associated genes were noted by the Reactome database [28] and yielded 129 pathways (Supplemental Table S1). Excitingly, 78 of the 129 enriched pathways had a *p*-value and an FDR less than 0.05 (Supplemental Table S1), representing strong confidence in the enrichment results from the skeletal class II malocclusion data. Based on the FDR, the top 20 enriched pathways (Table 2) were selected for further discussion regarding their functions related to bone and cartilage growth and development.

#### 3.1.3. The Skeletal Class II Malocclusion-Associated Genes Not Recognized by the Reactome Database

Two genes, *MSX1* and *MYO1H*, that are highly associated with skeletal class II malocclusion (appearing twice in the database search) were not indexed in the Reactome database. Consequently, the Uniprot database [29] was utilized to gain insights into the functions of *MSX1* and *MYO1H* instead (Table 3).

### 3.2. Skeletal Class III Malocclusion

#### 3.2.1. Gene Identification

Data mining resulted in the identification of 53 genes that were associated with skeletal class III malocclusion (Table 4): one gene, *matrilin 1* (*MATN1*), was indexed by four independent studies; six genes (*collagen type II alpha 1 chain* (*COL2A1*), *fibroblast growth factor receptor 2* (*FGFR2*), *lysine acetyltransferase 6B* (*KAT6B*), *myosin IH* (*MYO1H*), *plexin A2* (*PLXNA2*), and *SSX family member 2 interacting protein* (*SSX2IP*)) were investigated twice; and 46 genes once.

#### 3.2.2. Pathway Enrichment

Forty-four skeletal class III malocclusion-associated genes were recognized in the Reactome database, resulting in 425 enriched pathways (Supplemental Table S2). The enrichment results yielded 129 pathways with a *p*-value and an FDR less than 0.05 (Supplemental Table S2). Among them, the top 20 enriched pathways were selected for further discussion (Table 5).

#### 3.2.3. The Skeletal Class III Malocclusion-Associated Genes Not Recognized by the Reactome Database

Nine identified skeletal class III-associated genes, including *myosin heavy chain 1* (*MYH1*), *chromosome 1 open reading frame 167* (*C1orf167*), *homeobox C cluster* (*HOXC*), *NBPF member 8* (*NBPF8*), *NBPF member 9* (*NBPF9*), *transcription factor 21* (*TCF21*), *MYO1H*, *SSX2IP*, and *calneuron 1* (*CALN1*), were not found in the Reactome database. Notably, although 8 of these 9 genes appeared only once in previous GWAS studies, *MYO1H* was examined by two independent studies (Table 4). As above, the Uniprot database [29] was utilized to functionally annotate these skeletal class III malocclusion-associated genes that had not been indexed in the Reactome database (Table 6). However, functional annotation of *C1orf167*, *NBPF8*, and *NBPF9* failed in the Uniprot database, suggesting that knowledge of these genes is largely lacking.

### 3.3. Single Nucleotide Polymorphism (SNP) Analysis of The Genes Involved in Both Skeletal Class II and III Malocclusions

It is worth noting that four genes (*COL1A1*, *FGFR2*, *MATN1*, and *MYO1H*), were associated with both class II and class III skeletal malocclusions, indicating that these genes may play key regulatory roles in craniofacial growth, especially in the sagittal dimension. One possibility is that for each of these four genes, different SNPs may lead to a more active isoform or higher expression in one class malocclusion and a more inert isoform or less expression in the other. However, available GWAS studies [30,34,35,38,40,43,46,47,48,53,54] revealed that certain SNPs, including rs2249492 (*COL1A1*), rs11200014 (*FGFR2*), rs2162540 (*FGFR2*), rs10850110 (*MYO1H*), and rs3825393 (*MYO1H*), were shared by skeletal class II and class III malocclusions (Table 7), which cannot be explained by the hypothesis mentioned above. Surprisingly, most of the reported skeletal class II or class III malocclusion-associated SNPs are located in the introns of these four genes [74,75,76,77,78,79,80,81], indicating the importance of the noncoding regions of these DNA fragments in craniofacial development and skeletal malocclusion.

## 4. Discussion

### 4.1. FGFR2 and Related Pathways

*FGFR2*, a protein receptor for *FGFs*, affects osteoblasts’ proliferation, differentiation, and apoptosis, implicating it in bone growth [85]. Moreover, *FGFR2* mutations have previously been linked to bone development and growth diseases, such as Apert syndrome [86], a genetic syndrome characterized by untimely early fusion of the skull bones during development that displays skeletal class III malocclusion [87,88]. Noticeably, Apert syndrome can arise from the S252W mutation in *FGFR2* [88]. In this study, enrichment results showed that *FGFR2* is involved in 19 of the top 20 skeletal class III malocclusion-associated pathways (Table 5), echoing the assertion that this receptor and the associated FGFR pathway play a vital role in the sagittal disharmony of the maxillomandibular complex. Meanwhile, *FGFR2* was also found to be linked to skeletal class II malocclusion (Table 1) and appeared in 17 of the top 20 skeletal class II malocclusion-associated pathways (Table 2), suggesting that *FGFR2*’s influence is not limited to skeletal class III malocclusion but extends to skeletal class II malocclusion as well.

A variety of proteins have been identified as *FGFR2* regulators in bone cells. For instance, fibroblast growth factor receptor substrate 2 (FRS2) displays a bi-directional modulation of FGFR2 function: via direct binding, FRS2 induces FGFR2 degradation [89,90], while also enhancing FGFR2-related activation of mitogen-activated protein kinase 1 (MAPK3/ERK 1) [89,90] and thus orchestrating FGFR2’s bioactivity in osteoblastogenesis [89,90,91]. In addition, *FGFR2*’s expression is also negatively regulated by non-specific alkaline phosphatase (TNAP) [92], which is encoded by *ALPL* [93]—a recognized skeletal class III malocclusion-associated gene (Table 4) [45]. Moreover, in osteoblasts, the translational product of the gene *matrix metalloproteinase 14* (*MMP14*), termed MT-MMP, can prohibit the digestion of FGFR2 by ADAM metallopeptidase domain 9 (ADAM9) to preserve FGFR2’s function [94]. Interestingly, other members of the metalloproteinase family were also associated with skeletal malocclusions. For example, *ADAMTS9* is a skeletal class II malocclusion-associated gene (Table 1) [32,33], while *ADAMTS1*, *ADAMTSL1*, and *MMP13* [49,58,59] were associated with skeletal class III malocclusion (Table 4). Thus, the crosstalk between the metalloproteinase family with FGFR2 may be interesting for further investigation in the context of skeletal malocclusion development and progression.

Regarding FGFR2’s downstream signal transduction, “Phospholipase C-mediated cascade; FGFR2” is one of the enriched pathways shared by skeletal class II and III malocclusions (Table 2 and Table 5). In this pathway, phospholipase C-γ (PLCγ) is a substrate of FGFR and other receptors with tyrosine kinase activity [95]. Particularly, a previous study revealed that FGFR2 is responsible for PLCγ2 signaling activation in rat osteoblasts [96]. Furthermore, it is worth noting that PLCγ2 also promotes bone resorption by upregulating the expression of nuclear factor of activated T-cells, cytoplasmic 1 (NFATc1), a transcription factor that plays a central role in promoting osteoclastogenesis [97]. Consequently, impairment of the PLCγ2 pathway depresses osteoclastogenesis [98]. In addition, *NFATc1* is a skeletal class III malocclusion-associated gene (Table 4). Thus, the FGFR2→PLCγ2→NFATc1 signal axis may be one of the essential bone growth and development regulating pathways that modulate osteoblast and osteoclast activities and thus contribute to skeletal malocclusion establishment. Meanwhile, since more *FGFR2* SNPs were reported in skeletal class III malocclusion than in skeletal class II malocclusion, the influence of the FGFR2→PLCγ2→NFATc1 cassette may be more common and notable for skeletal class III malocclusion progression; however, further investigation targeting a population of greater diversity should be conducted to test this hypothesis.

Downregulated by FGFR2 [99], the “PI3K Cascade” is another skeletal class II and III malocclusion-associated pathway (Table 2 and Table 5). Previous studies showed that inhibition of PI3K/p70 S6K cascades increases osteoblastic differentiation induced by *bone morphogenetic protein 2* (*BMP2*) [100] while decreasing osteoclast activity [101]. These results suggest that the “PI3K Cascade” may govern bone growth and development concerning both osteoblasts and osteoclasts, while further research is needed to parse out how this pathway is associated with skeletal class II and class III malocclusions.

Interestingly, all reported *FGFR2* SNPs are located in introns (Table 7). As opposed to exons, which directly encode protein sequences, introns integrally regulate gene expression [102]. Some *FGFR2* SNPs, such as rs10736303, rs1078806, and rs2981578, were only detected in skeletal class III malocclusion (Table 7) [35], suggesting that these SNPs may be responsible for mandibular prognathism and/or maxillary retrognathism. Surprisingly, two *FGFR2* SNPs from the same intron, rs11200014 and rs2162540, are linked to both skeletal class II and III malocclusions (Table 7) [34,35]. It is difficult to explain the influence of these two *FGFR2* SNPs if *FGFR2* is considered as functioning in an isolated manner. One possible explanation is that after post-transcriptional modification, the respective introns are removed from the pre-mRNA and serve as miRNAs that regulate the expression of other skeletal malocclusion-associated genes, while the SNPs significantly alter their regulation targets and/or their effectiveness. Alternatively, these two intronic SNPs may make *FGFR2* expression more sensitive to other influences and thus increase the risk of abnormal skeletal malocclusions. These additional influences may not be limited to other growth factors or cytokines involved in bone and cartilage growth and development. It is also possible that these two intronic *FGFR2* SNPs indicate a scenario in which the affected bone is more vulnerable and sensitive to mechanical stimulations, such as those from the attached muscle tissue, as proposed by functional matrix theory [103,104].

### 4.2. Insulin Receptor Cascade

“Insulin receptor signaling cascade”, which was identified by pathway enrichment of the genes associated with skeletal class II and class III malocclusions (Table 2 and Table 5), is known to promote osteoblast differentiation and osteocalcin (OCN) secretion [105,106,107]. Moreover, deletion of the *Insulin-like growth factor 1 receptor (IGF1R)* gene in mouse osteoblasts led to a striking decrease in cancellous bone volume, connectivity, and trabecular number, accompanied by an increase in trabecular spacing, while the rate of mineralization of osteoid was significantly decreased [108]. This correlation suggests that the “Insulin receptor signaling cascade” is essential for coupling matrix biosynthesis to sustained mineralization, which is particularly important during the pubertal growth spurt when rapid bone formation and consolidation are required [108].

Both insulin receptor substrate 1 (IRS1) and insulin receptor substrate 2 (IRS2) are involved in the signal transduction from IGF1R to its downstream mediator RAC-alpha serine/threonine-protein kinase (Akt1) [109]. Previous studies demonstrate that IRS1 in osteoblasts is indispensable for maintaining bone turnover [110]. In contrast, IRS2 is more predominantly required for bone formation over bone resorption [111]. Indeed, Akune et al. found that IRS2 signaling is not essential for osteoclastic cells’ differentiation, functioning, or survival [111]. Therefore, balanced IRS1/IRS2 signal transduction may contribute to the foundation of normal bone development and growth, while breaking the equilibrium of the “Insulin receptor signaling cascade” may trigger the formation of skeletal abnormalities, including skeletal class II and III malocclusions.

Because they are both connected to the PI3K→Akt axis, a complex interaction exists between FGF-related signal transduction and the “Insulin receptor signaling cascade” [99,109,112]. For example, not only is IRS1 involved in the “Insulin receptor signaling cascade” [109], but it is also a part of the “PI3K cascade” [113], and thus its influence may also extend to osteoclasts. In addition, stimulating the “Insulin receptor signaling cascade” could activate the parathyroid hormone (PTH) type 1 receptor (PTH1R) in osteoblasts/osteocytes to enhance the osteoblast-to-osteocyte transition [114]. Thus, the “Insulin receptor signaling cascade” may act as an intermediate coordinator of various signal pathways to integrate functions of theirs that are related to bone growth and development regulation and may warrant further investigation regarding its association with craniofacial sagittal discrepancy.

### 4.3. Runt-Related Transcription Factor 2 (RUNX2) and Notch Receptor 3 (NOTCH3)

As a well-known, predominant regulator of bone and cartilage growth and development [115,116], *runt-related transcription factor 2* (*RUNX2*) promotes the activity of *FGFR2* and *FGFR3*, leading to the proliferation of pre-osteoblastic cells [117]. Considering that *RUNX2* promotes the differentiation of secondary chondrocytes and the formation of the cartilage of mandibular condyles [118], genetic variations that are more likely to have a negative influence on the *RUNX2* gene may more frequently lead to mandibular retrognathism and thus induce skeletal class II malocclusion. This hypothesis is supported by previous animals studies that have shown that *RUNX2*-deficiency in mice resulted in a lack of mandibular condylar cartilage and mandibular bone [119]. In the current study, the *RUNX2* gene was identified as the seed for three skeletal class II malocclusion-associated enriched pathways, as expected (Table 2).

On the other hand, the NOTCH signaling pathway was associated with skeletal class III malocclusion in the current study (Table 5), which is in agreement with previous research that demonstrates that a reduction in NOTCH3 signaling promoted osteogenic differentiation of mesenchymal stem stems located in a certain part of the mandible [120].

Together, the class-specific pathway identification of *RUNX2* and *NOTCH3* establishes confidence in the current skeletal malocclusion-associated enrichment.

### 4.4. Muscle-Related Genes with Skeletal Class II and III Malocclusions

#### 4.4.1. Functional Matrix Theory

Excitingly, some skeletal class II and III malocclusion-associated genes identified in the current study have muscle-related functional annotations (Table 1 and Table 4). One possible explanation for this is functional matrix theory, also called functional matrix hypothesis [103,104].

Functional matrix theory proposes that the growth and development of bone, including qualities such as attained length and width, are largely influenced by other body components [103,104]. For example, changes in the skeleton are not fully promoted by the genetic makeup of the bones but also rely on other biological components, particularly the skeletal muscles that belong to the periosteal category of the functional matrix [103]. Skeletal muscle activity could generate endogenous electrical fields that might orchestrate bone growth and development through various mechanisms [103]. On the other hand, the epigenetic event of muscle contraction may extend to bone cells’ genomes and thus modulate bone growth and development [121]. Therefore, as demonstrated in the current study (Table 1 and Table 4), although genes that regulate skeletal muscle generation and activities may not be directly involved in bone and cartilage growth and development, a number of them nevertheless play a role in the growth and development of the maxilla and mandible, influencing characteristics such as length, and consequently are involved in the development of skeletal class II and III malocclusions. On the other hand, SNPs found in genes associated with bone and cartilage growth and development, either in introns or exons, may cause the gene isoform to be more sensitive to intracellular mechanotransduction and/or more vulnerable to muscle contraction-initiated epigenetic modification, and thus contribute to skeletal malocclusion determination.

#### 4.4.2. Myosin Heavy Chain (MYH) Genes

Several *MYH* genes were previously associated with both types of malocclusions (Table 1 and Table 4). In particular, higher expression levels of *MYH3*, *MYH6*, and *MYH7* were detected in patients with mandibular retrognathism than in those with mandibular prognathism, while no significant difference was found between these two populations in regard to *MYH1*, *MYH2*, and *MYH8* expression levels [44]. Thus, it is safe to say that the MYH proteins could be future research targets for craniofacial skeletal sagittal growth prediction and modification. At the same time, further investigations are warranted to determine whether, which of, and how the MYH molecules are involved in skeletal malocclusion establishment and if and what characters were overlooked in these previous investigations.

#### 4.4.3. Other Muscle Function Genes

*Histone deacetylase 4* (*HDAC4*) was found to be more highly expressed in the masseter muscle of patients with skeletal class III malocclusion than in patients with skeletal class II malocclusion [51]. In addition, two independent studies revealed higher expression of *lysine acetyltransferase 6B* (*KAT6B*) in the masseter muscle of skeletal class III malocclusion patients than in the same tissue type belonging to skeletal class II malocclusion patients, thereby indicating that *KAT6B* may be associated with mandibular prognathism [51,52]. Clearly, these genes could also be candidates for predicting patients’ expected development of skeletal malocclusion and their clinical correction prognoses in the future.

It is important to note that GWAS can only indicate the association between identified genes and a targeted clinical condition, while functional validation at the molecular level is a prerequisite for forming any conclusions. As stated above, current knowledge of the functions of the skeletal class II and III-associated genes in the musculoskeletal system is extensive. However, the specific impacts of these genes on the craniofacial region are largely unknown. We hope that the current review and the conducted pathway enrichment can provide clues about the potential relationships among the identified genes and identify which genes and pathways can be prioritized for functional validation in future studies. We would also like to emphasize that the current review focused on the sagittal dimension, which is a crucial dimension in orthodontic diagnosis and treatment planning. Because craniofacial structure develops three-dimensionally, further evaluation of the genes involved in the vertical and transverse growth and development of the craniofacial structure is also needed.

## 5. Conclusions

By reviewing and reanalyzing available human studies, 19 genes were found to be associated with skeletal class II malocclusion and 53 genes with skeletal class III malocclusion. Using the Reactome database, most of these genes were enriched in pathways related to bone and cartilage regulation and growth, as expected. Interestingly, multiple muscle-related genes and pathways have also been identified, which aligns with functional matrix theory in that the muscles surrounding the jaws may also contribute to the development of these two types of malocclusions. In addition, several SNPs were associated with both skeletal class II and III malocclusions, with the majority of these SNPs being located in the introns of their respective genes. It is possible that these SNPs result in a more sensitive isoform of the respective genes in response to factors associated with the surrounding microenvironment, such as mechanotransductive stimulation and/or pre- and post-translational regulations that might include epigenetic modification and miRNA-level interaction. Although multiple confirmation studies must be conducted, we hope our current study can provide a relatively panoramic view of the current understanding of skeletal class II and III malocclusions at the genetic level and thus potentially help dental care providers make more accurate craniofacial sagittal growth predictions and treatment strategy selections based on the patients’ genetic profiles in the future.

## Figures and Tables

**Table 1:** The list of identified skeletal class II malocclusion-associated genes.

Frequency of Gene in Search	Name of Gene		Reference(s)
2	*ACTN3*	*actinin alpha 3*	[30,31]
	*ADAMTS9*	*ADAM metallopeptidase with thrombospondin type 1 motif 9*	[32,33]
	*FGFR2*	*fibroblast growth factor receptor 2*	[34,35]
	*MSX1*	*msh homeobox 1*	[36,37]
	*MYO1H*	*myosin IH*	[30,38]
1	*BMP2*	*bone morphogenetic protein 2*	[39]
	*COL1A1*	*collagen type I alpha 1 chain*	[40]
	*COL11A1*	*collagen type XI alpha 1 chain*	[40]
	*CYP24A1*	*cytochrome P450 family 24 subfamily A member 1*	[41]
	*CYP27B1*	*cytochrome P450 family 27 subfamily B member 1*	[41]
	*EDN1*	*endothelin 1*	[34]
	*GLI3*	*GLI family zinc finger 3*	[42]
	*MATN1*	*matrilin 1*	[43]
	*MYH3*	*myosin heavy chain 3*	[44]
	*MYH6*	*myosin heavy chain 6*	[44]
	*MYH7*	*myosin heavy chain 7*	[44]
	*PTH*	*parathyroid hormone*	[41]
	*RUNX2*	*RUNX family transcription factor 2*	[39]
	*VDR*	*vitamin D receptor*	[41]

**Table 2:** The top 20 enriched skeletal class II malocclusion-associated pathways.

Pathway Identifier	Pathway Name	Entities *p* Value	Entities FDR	Submitted Entities Found
R-HSA-1839126	FGFR2 mutant receptor activation	1.46 × 10^−10^	1.92 × 10^−8^	*FGFR2*
R-HSA-8851708	Signaling by FGFR2 IIIa TM	7.82 × 10^−10^	3.72 × 10^−8^	*FGFR2*
R-HSA-5655253	Signaling by FGFR2 in disease	8.65 × 10^−10^	3.72 × 10^−8^	*FGFR2*
R-HSA-8940973	RUNX2 regulates osteoblast differentiation	4.41 × 10^−9^	1.41 × 10^−7^	*COL1A1*; *RUNX2*; *GLI3*
R-HSA-8878166	Transcriptional regulation by RUNX2	5.99 × 10^−9^	1.41 × 10^−7^	*COL1A1*; *BMP2*; *RUNX2*; *GLI3*
R-HSA-1226099	Signaling by FGFR in disease	6.72 × 10^−9^	1.41 × 10^−7^	*FGFR2*
R-HSA-8941326	RUNX2 regulates bone development	1.41 × 10^−8^	2.54 × 10^−7^	*COL1A1*; *RUNX2*; *GLI3*
R-HSA-5654221	Phospholipase C-mediated cascade; FGFR2	1.27 × 10^−7^	2.03 × 10^−6^	*FGFR2*
R-HSA-190241	FGFR2 ligand binding and activation	1.48 × 10^−7^	2.07 × 10^−6^	*FGFR2*
R-HSA-5654695	PI-3K cascade:FGFR2	2.97 × 10^−7^	3.87 × 10^−6^	*FGFR2*
R-HSA-5654699	SHC-mediated cascade:FGFR2	3.81 × 10^−7^	4.19 × 10^−6^	*FGFR2*
R-HSA-5654700	FRS-mediated FGFR2 signaling	4.29 × 10^−7^	4.29 × 10^−6^	*FGFR2*
R-HSA-5654727	Negative regulation of FGFR2 signaling	8.99 × 10^−7^	8.90 × 10^−6^	*FGFR2*
R-HSA-5654696	Downstream signaling of activated FGFR2	9.89 × 10^−7^	8.90 × 10^−6^	*FGFR2*
R-HSA-109704	PI3K Cascade	3.53 × 10^−6^	2.82 × 10^−5^	*FGFR2*
R-HSA-112399	IRS-mediated signalling	5.52 × 10^−6^	3.86 × 10^−5^	*FGFR2*
R-HSA-2428928	IRS-related events triggered by IGF1R	6.97 × 10^−6^	4.18 × 10^−5^	*FGFR2*
R-HSA-2428924	IGF1R signaling cascade	8.23 × 10^−6^	4.35 × 10^−5^	*FGFR2*
R-HSA-74751	Insulin receptor signalling cascade	8.23 × 10^−6^	4.35 × 10^−5^	*FGFR2*
R-HSA-2404192	Signaling by Type 1 Insulin-like Growth Factor 1 Receptor (IGF1R)	8.69 × 10^−6^	4.35 × 10^−5^	*FGFR2*

**Table 3:** Functional annotation of the skeletal class II malocclusion-associated genes not recognized by the Reactome database.

Gene Name	Function
*MSX1*	activation of meiosis, anterior/posterior pattern specification, BMP signaling pathway involved in heart development, bone morphogenesis, cardiac conduction system development, cartilage morphogenesis, cell morphogenesis, cellular response to nicotine, embryonic forelimb morphogenesis, embryonic hindlimb morphogenesis, embryonic morphogenesis, embryonic nail plate morphogenesis, epithelial to mesenchymal transition involved in endocardial cushion formation, face morphogenesis, in utero embryonic development, mammary gland epithelium development, mesenchymal cell proliferation, midbrain development, middle ear morphogenesis, muscle organ development, negative regulation of apoptotic process, negative regulation of cell growth, negative regulation of cell population proliferation, negative regulation of striated muscle cell differentiation, negative regulation of transcription regulatory region DNA binding, odontogenesis of dentin-containing tooth, pituitary gland development, positive regulation of BMP signaling pathway, positive regulation of DNA damage response, signal transduction by p53 class mediator, positive regulation of intrinsic apoptotic signaling pathway by p53 class mediator, positive regulation of mesenchymal cell apoptotic process, protein localization to nucleus, protein stabilization, regulation of odontogenesis, regulation of transcription by RNA polymerase II, central roof of mouth development, signal transduction involved in regulation of gene expression, stem cell differentiation
*MYO1H*	actin filament organization, vesicle transport along actin filament

**Table 4:** The list of identified skeletal class III malocclusion-associated genes.

Frequency of Gene in Search	Name of Gene		Reference(s)
4	*MATN1*	*matrilin 1*	[45,46,47,48]
2	*COL2A1*	*collagen type II alpha 1 chain*	[49,50]
	*FGFR2*	*fibroblast growth factor receptor 2*	[34,35]
	*KAT6B*	*lysine acetyltransferase 6B*	[51,52]
	*MYO1H*	*myosin IH*	[53,54]
	*PLXNA2*	*plexin A2*	[49,55]
	*SSX2IP*	*SSX family member 2 interacting protein*	[56,57]
1	*ADAMTS1*	*ADAM metallopeptidase with thrombospondin type 1 motif 1*	[58]
	*ADAMTSL1*	*ADAMTS like 1*	[59]
	*ALPL*	*alkaline phosphatase, biomineralization associated*	[45]
	*ARHGAP21*	*Rho GTPase activating protein 21*	[60]
	*BEST3*	*bestrophin 3*	[61]
	*C1orf167*	*chromosome 1 open reading frame 167*	[62]
	*CALN1*	*calneuron 1*	[56]
	*COL1A1*	*collagen type I alpha 1 chain*	[34]
	*DUSP6*	*dual specificity phosphatase 6*	[63]
	*EP300*	*E1A binding protein p300*	[64]
	*EPB41*	*erythrocyte membrane protein band 4.1*	[65]
	*ERLEC1*	*endoplasmic reticulum lectin 1*	[66]
	*EVC*	*EvC ciliary complex subunit 1*	[67]
	*EVC2*	*EvC ciliary complex subunit 2*	[67]
	*FGF12*	*fibroblast growth factor 12*	[68]
	*FGF20*	*fibroblast growth factor 20*	[68]
	*FGF23*	*fibroblast growth factor 23*	[69]
	*FGF3*	*fibroblast growth factor 3*	[70]
	*FGFR1*	*fibroblast growth factor receptor 1-A*	[68]
	*FOXO3*	*forkhead box O3*	[71]
	*GHR*	*growth hormone receptor*	[72]
	*GLI2*	*GLI family zinc finger 2*	[42]
	*HDAC4*	*histone deacetylase 4*	[51]
	*HOXC*	*homeobox C cluster*	[49]
	*HSPG2*	*heparan sulfate proteoglycan 2*	[45]
	*IGF1*	*insulin like growth factor 1*	[49]
	*JAG1*	*jagged canonical Notch ligand 1*	[64]
	*LTBP2*	*latent transforming growth factor beta binding protein 2*	[73]
	*MMP13*	*matrix metallopeptidase 13*	[49]
	*MYH1*	*myosin heavy chain 1*	[71]
	*MYH8*	*myosin heavy chain 8*	[71]
	*NBPF8*	*NBPF member 8*	[62]
	*NBPF9*	*NBPF member 9*	[62]
	*NCOR2*	*nuclear receptor corepressor 2*	[64]
	*NFATC1*	*nuclear factor of activated T cells 1*	[71]
	*NOTCH3*	*notch receptor 3*	[64]
	*NOTCH4*	*notch receptor 4*	[64]
	*NUMB*	*NUMB endocytic adaptor protein*	[64]
	*PSEN2*	*presenilin 2*	[64]
	*RASA2*	*RAS p21 protein activator 2*	[56]
	*RORA*	*RAR related orphan receptor A*	[56]
	*SMAD6*	*SMAD family member 6*	[39]
	*TBX5*	*T-box transcription factor 5*	[34]
	*TCF21*	*transcription factor 21*	[56]
	*TGFB3*	*transforming growth factor beta 3*	[73]
	*WNT3A*	*Wnt family member 3A*	[39]

**Table 5:** The top 20 enriched skeletal class III malocclusion-associated pathways.

Pathway Identifier	Pathway Name	Entities *p* Value	Entities FDR	Submitted Entities Found
R-HSA-5663202	Diseases of signal transduction by growth factor receptors and second messengers	3.89 × 10^−15^	1.84 × 10^−12^	*HDAC4*; *JAG1*; *WNT3A*; *PSEN2*; *FOXO3*; *DUSP6*; *FGF3*; *NCOR2*; *ERLEC1*; *FGF20*; *EP300*; *FGF23*; *FGFR2*; *FGFR1*
R-HSA-1226099	Signaling by FGFR in disease	1.64 × 10^−14^	3.89 × 10^−12^	*FGF20*; *FGF23*; *FGFR2*; *FGF3*; *FGFR1*
R-HSA-1839126	FGFR2 mutant receptor activation	1.61 × 10^−12^	2.54 × 10^−10^	*FGF20*; *FGF23*; *FGFR2*; *FGF3*
R-HSA-2428928	IRS-related events triggered by IGF1R	3.15 × 10^−12^	3.72 × 10^−10^	*FGF20*; *IGF1*; *FGF23*; *FGFR2*; *FGF3*; *FGFR1*
R-HSA-2428924	IGF1R signaling cascade	4.77 × 10^−12^	4.31 × 10^−10^	*FGF20*; *IGF1*; *FGF23*; *FGFR2*; *FGF3*; *FGFR1*
R-HSA-2404192	Signaling by Type 1 Insulin-like Growth Factor 1 Receptor (IGF1R)	5.45 × 10^−12^	4.31 × 10^−10^	*FGF20*; *IGF1*; *FGF23*; *FGFR2*; *FGF3*; *FGFR1*
R-HSA-5655253	Signaling by FGFR2 in disease	2.24 × 10^−11^	1.16 × 10^−9^	*FGF20*; *FGF23*; *FGFR2*; *FGF3*
R-HSA-109704	PI3K Cascade	2.24 × 10^−11^	1.16 × 10^−9^	*FGF20*; *FGF23*; *FGFR2*; *FGF3*; *FGFR1*
R-HSA-112399	IRS-mediated signalling	6.06 × 10^−11^	2.61 × 10^−9^	*FGF20*; *FGF23*; *FGFR2*; *FGF3*; *FGFR1*
R-HSA-5654221	Phospholipase C-mediated cascade; FGFR2	7.22 × 10^−11^	2.82 × 10^−9^	*FGF20*; *FGF23*; *FGFR2*; *FGF3*
R-HSA-190241	FGFR2 ligand binding and activation	9.46 × 10^−11^	3.41 × 10^−9^	*FGF20*; *FGF23*; *FGFR2*; *FGF3*
R-HSA-74751	Insulin receptor signalling cascade	1.48 × 10^−10^	4.88 × 10^−9^	*FGF20*; *FGF23*; *FGFR2*; *FGF3*; *FGFR1*
R-HSA-5654695	PI-3K cascade:FGFR2	3.18 × 10^−10^	9.85 × 10^−9^	*FGF20*; *FGF23*; *FGFR2*; *FGF3*
R-HSA-5654699	SHC-mediated cascade:FGFR2	4.88 × 10^−10^	1.42 × 10^−8^	*FGF20*; *FGF23*; *FGFR2*; *FGF3*
R-HSA-5654700	FRS-mediated FGFR2 signaling	5.99 × 10^−10^	1.62 × 10^−8^	*FGF20*; *FGF23*; *FGFR2*; *FGF3*
R-HSA-157118	Signaling by NOTCH	6.57 × 10^−10^	1.71 × 10^−8^	*NCOR2*; *HDAC4*; *NOTCH3*; *JAG1*; *NOTCH4*; *PSEN2*; *NUMB*; *EP300*
R-HSA-2219528	PI3K/AKT Signaling in Cancer	8.88 × 10^−10^	2.13 × 10^−8^	*FGF20*; *FOXO3*; *FGF23*; *FGFR2*; *FGF3*; *FGFR1*
R-HSA-2219530	Constitutive Signaling by Aberrant PI3K in Cancer	1.79 × 10^−9^	4.11 × 10^−8^	*FGF20*; *FGF23*; *FGFR2*; *FGF3*; *FGFR1*
R-HSA-74752	Signaling by Insulin receptor	1.95 × 10^−9^	4.30 × 10^−8^	*FGF20*; *FGF23*; *FGFR2*; *FGF3*; *FGFR1*
R-HSA-5654727	Negative regulation of FGFR2 signaling	2.16 × 10^−9^	4.54 × 10^−8^	*FGF20*; *FGF23*; *FGFR2*; *FGF3*

**Table 6:** Functional annotation of the skeletal class III malocclusion-associated genes not recognized by the Reactome database.

Gene Name	Function
*MYH1*	muscle contraction
*C1orf167*	-
*HOXC*	anatomical structure morphogenesis; anterior/posterior pattern specification; hair follicle development; nail development; positive regulation of transcription, DNA-templated; regulation of transcription by RNA polymerase II, tongue morphogenesis
*NBPF8*	-
*NBPF9*	-
*TCF21*	branching involved in ureteric bud morphogenesis, branchiomeric skeletal muscle development, bronchiole development, developmental process, diaphragm development, embryonic digestive tract morphogenesis, epithelial cell differentiation, gland development, glomerulus development, kidney development, lung alveolus development, lung morphogenesis, lung vasculature development, metanephric glomerular capillary formation, metanephric mesenchymal cell differentiation, morphogenesis of a branching structure, negative regulation of androgen receptor signaling pathway, negative regulation of transcription by RNA polymerase II, positive regulation of transcription by RNA polymerase II, regulation of transcription by RNA polymerase II, reproductive structure development, respiratory system development, roof of mouth development, Sertoli cell differentiation, sex determination, spleen development, ureteric bud development, vasculature development
*MYO1H*	actin filament organization, vesicle transport along actin filament
*SSX2IP*	cell adhesion, centrosome cycle, cilium assembly, intraciliary transport involved in cilium assembly, regulation of cell motility, regulation of Rac protein signal transduction
*CALN1*	Negatively regulates Golgi-to-plasma membrane trafficking by interacting with PI4KB and inhibiting its activity. May play a role in the physiology of neurons and is potentially important in memory and learning.

**Table 7:** SNPs of the genes associated with both skeletal class II and III malocclusions.

Gene Name	Class II				Class III			
	SNP#	Location	Nucleotide Change	Classification	SNP#	Location	Nucleotide Change	Classification
*COL1A1*	rs2249492 [40]	Intron	C > G/C > T [74]	-	rs2249492 [34]	Intron	C > G/C > T [74]	-
*FGFR2*	rs11200014 [34]	Intron	G > A/G > T [75]	-	rs11200014 [35]	Intron	G > A/G > T [75]	-
	rs2162540 [34,35]	Intron	C > A/C > T [76]	-	rs2162540 [34]	Intron	C > A/C > T [76]	-
					rs10736303 [35]	Intron	G > A/G > T [77]	-
					rs1078806 [35]	Intron	A > G/A > T [78]	-
					rs2981578 [35]	Intron	C > A/C > T [79]	-
*MATN-1*	rs1149042 [43]	Intron	T > G [80]	-	rs20566 [46,47,48]	Exon	A > G/A > T [82]	Synonymous Variant
					rs1065755 [46,47]	Exon	C > T [83]	Synonymous Variant
*MYO1H*	rs10850110 [30]	Intron (promoter)	G > A/G > T [81]	-	rs10850110 [53]	Intron (promoter)	G > A, G > T [81]	-
	rs3825393 [38]	Exon	T > C/T > G [84]	Missense mutation	rs3825393 [54]	Exon	T > C/T > G [84]	Missense mutation

## Data Availability

The data presented in this study are contained within this article and Supplementary Materials.

## Supplementary Materials

The following are available online at https://www.mdpi.com/article/10.3390/ijms222313037/s1.
